# Gut microbiota and postoperative delirium: mechanistic integration of the “gut-liver-anesthesia axis” in the context of liver disease and perioperative intervention strategies

**DOI:** 10.3389/fmed.2026.1832034

**Published:** 2026-05-20

**Authors:** Rui Han, Yuanyuan Song, Yu Wang, Yuhao Liu, Xiaowen Zhang, Zihao Deng, Lingyi Xia, Mao-Lin Zhong

**Affiliations:** 1 The First Clinical Medical College, Gannan Medical University, Ganzhou, China; 2 Department of Anesthesiology, The First Affiliated Hospital of Gannan Medical University, Ganzhou, China; 3 Anesthesia Key Laboratory of Gannan Medical University, Ganzhou, China

**Keywords:** postoperative delirium, gut microbiota, gut-brain axis, gut-liver axis, liver disease, anesthesia, perioperative intervention

## Abstract

Postoperative delirium (POD) is a prevalent and clinically significant complication among older surgical patients. Despite its high incidence, the underlying pathogenesis remains incompletely understood, and effective targeted therapeutic strategies are lacking. As the largest microecosystem in the human body, the gut microbiota has been increasingly recognized for its potential role in modulating central nervous system (CNS) function and systemic inflammatory responses through the gut–brain and gut–liver axes. Accumulating evidence suggests that alterations in gut microbial composition (gut dysbiosis) may be associated with the initiation and progression of POD. Patients with pre-existing liver disease appear to be particularly vulnerable, possibly due to baseline disturbances in gut microbiota, impaired intestinal barrier function, and systemic immune dysregulation. In addition, exposure to anesthetic agents and the physiological stress of surgery may further perturb intestinal microecology, potentially contributing to a self-reinforcing cycle within a proposed gut-liver-anesthesia axis. In this narrative review, we provide a targeted synthesis of current mechanistic and clinical evidence linking the gut microbiota to POD. We further explore the potential interactions among gut dysbiosis, liver dysfunction, and perioperative anesthetic factors, with a focus on their implications for neurocognitive outcomes. Finally, we summarize emerging microbiota-targeted perioperative interventions, while emphasizing that the current evidence remains limited, heterogeneous, and largely hypothesis-generating rather than directly translatable to clinical practice.

## 1 Introduction

Despite the considerable clinical burden imposed by POD, its pathogenesis remains incompletely defined. Current evidence implicates neuroinflammation, neurotransmitter dysregulation, blood–brain barrier (BBB) disruption, and Alzheimer’s disease–associated neuropathological alterations—such as phosphorylated tau deposition-as potential contributing mechanisms (1, 2). Nevertheless, these processes do not fully explain the marked interindividual variability and clinical heterogeneity characteristic of POD, suggesting that additional upstream regulatory factors remain to be elucidated.

The gut microbiota constitutes a complex and dynamic microecosystem within the human intestinal tract. In recent years, the concept of the gut-brain axis has highlighted that gut microbes not only participate in nutrient metabolism and maintenance of mucosal immune homeostasis but may also engage in bidirectional communication with the CNS via neuroendocrine, immune, and metabolite-mediated pathways (3, 4). Experimental studies have demonstrated that microbial imbalance may increase BBB permeability, activate microglia, exacerbate hippocampal neuroinflammation, and impair cognitive performance (5). Preliminary findings from our group further indicate that aged mice subjected to anesthesia and surgery exhibit pronounced shifts in microbial composition accompanied by delirium-like behaviors and cognitive decline; notably, supplementation with Lactobacillus or multi-strain probiotics partially attenuated these effects (6). While these observations are primarily derived from preclinical models, they provide a biologically plausible framework supporting the hypothesis that perioperative perturbations of intestinal microecology may influence POD risk.

The liver and intestine are anatomically and functionally interconnected through the portal venous circulation, forming the gut-liver axis (7). Patients with liver disease frequently exhibit gut dysbiosis, impaired intestinal barrier function, and bacterial translocation—features that may influence not only hepatic disease progression but also systemic inflammation and brain function via a gut-liver-brain axis (8). With the increasing global prevalence of metabolic dysfunction–associated fatty liver disease (MASLD) and alcohol-related liver disease, the number of surgical patients with coexisting hepatic impairment is rising (9, 10). These individuals appear to have an increased susceptibility to POD, and their baseline disturbances in microecological balance, barrier integrity, and metabolic function provide a clinically relevant context for exploring potential mechanisms underlying POD.

In this narrative review, we integrate recent advances in gut microbiology, hepatology, and anesthesiology to explore potential mechanisms linking the gut microbiota to POD. We specifically examine the relevance of interactions among gut dysbiosis, liver dysfunction, and perioperative anesthetic factors, and summarize emerging microbiota-targeted perioperative strategies. Importantly, this review is intended to provide a conceptual synthesis of current evidence and to identify areas requiring further investigation, rather than to offer definitive clinical recommendations.

Although the term “gut-liver-anesthesia axis” has been previously mentioned in the literature (11), its application in the context of POD remains limited. To our knowledge, few studies have systematically integrated gut microbiota dynamics, liver function, and anesthetic exposure into a unified conceptual framework for perioperative neurocognitive disorders. In this review, we extend this concept in two respects. First, we focus specifically on patients with pre-existing chronic liver disease, a population characterized by baseline dysbiosis, impaired intestinal barrier function, chronic systemic inflammation, and reduced capacity for neurotoxin clearance, all of which may increase vulnerability to POD. Second, we incorporate emerging insights into bidirectional pharmacomicrobiomic interactions, including microbial modulation of hepatic drug-metabolizing pathways via nuclear receptors such as pregnane X receptor (PXR) and farnesoid X receptor (FXR), as well as the differential effects of anesthetic agents on gut microbial composition and barrier integrity.

Collectively, this integrative framework is intended to provide a more comprehensive and hypothesis-generating perspective on the potential mechanisms underlying POD, particularly in high-risk populations, while acknowledging that further experimental and clinical validation is required.

### 1.1 Literature search strategy

This article is a narrative review based on the authors’ domain expertise and a targeted literature search, rather than a formal systematic review. Relevant studies were identified through searches of PubMed, Web of Science, and Google Scholar for articles published up to March 2026, using combinations of keywords including “postoperative delirium,” “gut microbiota,” “gut-brain axis,” “gut-liver axis,” “liver disease,” “anesthesia,” “perioperative intervention,” “probiotics,” “fecal microbiota transplantation,” and “pharmacomicrobiomics.” Study selection was guided by relevance to the conceptual framework of the gut-liver-anesthesia axis. Priority was given to high-quality clinical studies, mechanistic animal research, and recent review articles where appropriate. In addition, reference lists of selected articles were manually screened to identify further relevant publications.

Given the narrative nature of this review, no predefined inclusion or exclusion criteria were applied, and no formal quality assessment, quantitative synthesis, or PRISMA flow diagram was conducted. The objective was to provide a focused and concept-driven synthesis of representative evidence rather than an exhaustive or systematic evaluation of the literature.

## 2 Gut microbiota and the gut-brain-axis: theoretical basis

### 2.1 Composition and function of the gut microbiota

The gut microbiota of healthy adults is predominantly composed of the phyla Firmicutes, Bacteroidetes, Actinobacteria, and Proteobacteria, with Firmicutes and Bacteroidetes typically representing the most abundant taxa (12). Functionally specialized bacterial groups, particularly short-chain fatty acid (SCFA)-producing families such as Lachnospiraceae and Ruminococcaceae, ferment dietary fiber to generate metabolites including butyrate, propionate, and acetate. These SCFAs play an important role in supporting colonic epithelial energy metabolism and maintaining intestinal barrier integrity (13).

Beyond its role in digestion and nutrient absorption, the gut microbiota is involved in vitamin synthesis, bile acid metabolism, and the modulation of xenobiotic processing. The resident microbial community also contributes to colonization resistance against enteric pathogens through mechanisms such as competitive exclusion and the production of antimicrobial compounds (14). Furthermore, the microbiota plays a critical role in the development and functional maturation of the host immune system; germ-free animals exhibit profound immunological deficiencies (15), highlighting the close relationship between microecological homeostasis and systemic inflammatory regulation.

### 2.2 Anatomical and physiological basis of the gut-brain axis

The gut-brain axis represents a complex and bidirectional communication network linking the gastrointestinal tract with the CNS. This interaction is mediated through multiple interconnected pathways, including neural, endocrine, immune, and metabolic mechanisms. Through these routes, signals derived from the gut microbiota may influence neurophysiological processes, while central regulatory mechanisms may in turn modulate gastrointestinal function and microbial composition (3, 4).

#### 2.2.1 Neural pathway

Afferent vagal fibers innervating the intestinal mucosa can detect microbial metabolites—including SCFAs, serotonin, and bacterial cell wall components—via specialized enteroendocrine and neuropod cells that form synapse-like connections with vagal terminals (3, 16). Activation of these afferent pathways transmits signals to the nucleus tractus solitarius and subsequently to limbic and cortical regions, potentially influencing mood, cognition, and stress responsiveness. Conversely, efferent vagal signaling modulates gut motility, secretion, and local immune activity through the enteric nervous system.

In the perioperative context, surgical stress and certain anesthetic agents may reduce vagal tone, potentially weakening the cholinergic anti-inflammatory pathway and contributing to systemic and neuroinflammatory responses. Although this mechanism is biologically plausible, its direct role in POD remains to be further clarified.

#### 2.2.2 Endocrine pathway

Enteroendocrine cells distributed throughout the intestinal epithelium respond to microbial metabolites by secreting gut-derived hormones such as cholecystokinin, glucagon-like peptide-1 (GLP-1), and peptide YY (PYY). After entering the circulation, these peptides may influence CNS function either by accessing regions with a permeable BBB or by activating receptors on vagal afferent pathways. For example, GLP-1 receptor signaling has been associated with the modulation of neuroinflammatory responses and synaptic plasticity.

Accordingly, alterations in gut microbial composition may affect enteroendocrine signaling profiles, which could, in turn, influence perioperative neurocognitive function. However, the extent to which these mechanisms contribute to POD in clinical settings remains to be further established.

#### 2.2.3 Immune pathway

The gut-associated lymphoid tissue (GALT) represents the largest immune compartment in the body, and its homeostasis is continuously shaped by microbial signals. Microbial components [e.g., lipopolysaccharide (LPS), flagellin] and metabolites (e.g., SCFAs, secondary bile acids) contribute to the regulation of immune balance, including the equilibrium between pro-inflammatory (Th17) and anti-inflammatory (regulatory T cell, Treg) responses, as well as the activation state of intestinal macrophages and dendritic cells (17).

Under conditions of impaired intestinal barrier function-commonly observed in chronic liver disease and potentially exacerbated by anesthesia and surgical stress-microbial products and immune mediators may translocate into the systemic circulation, contributing to a state of low-grade inflammation. Circulating cytokines and activated immune cells may subsequently influence CNS function via the BBB or circumventricular regions, promoting microglial activation and neuroinflammatory responses. While these processes are widely considered relevant to POD pathogenesis, their precise causal contributions in clinical settings remain to be fully established.

#### 2.2.4 Metabolite pathway

Gut microbial metabolites represent a diverse group of bioactive molecules linking microbial activity with host physiological processes. SCFAs (acetate, propionate, butyrate), produced through the fermentation of dietary fiber, contribute to the maintenance of intestinal barrier integrity, modulation of systemic immune responses, and can cross the BBB to influence microglial maturation and neuronal function (18, 19).

Secondary bile acids, generated via microbial deconjugation and dehydroxylation, act on nuclear receptors such as FXR and the G protein-coupled receptor TGR5, thereby participating in the regulation of hepatic metabolism and inflammatory signaling within the CNS. In addition, tryptophan-derived metabolites (e.g., indole derivatives, kynurenine) produced through microbial metabolism can activate pathways such as the aryl hydrocarbon receptor, influencing neuroinflammation and neurotransmitter balance.

Alterations in these metabolite profiles-commonly observed in liver disease and potentially exacerbated by surgical stress and anesthetic exposure-may represent an important mechanistic link between gut dysbiosis and POD. However, further studies are required to clarify their specific roles and clinical relevance in the perioperative setting.

### 2.3 Gut dysbiosis and neurocognitive disorders

A growing body of clinical and preclinical evidence supports an association between neuropsychiatric disorders and alterations in gut microbial composition (20–23). In the perioperative context, studies in aged animal models have shown that anesthesia- or surgery-induced shifts in microbial ecology occur in parallel with the development of cognitive impairment, a phenotype that can be partially ameliorated by probiotic supplementation (6).

These findings provide biologically plausible evidence for the involvement of gut microbiota in POD pathophysiology. In addition, a recent review has summarized the interactions between gut dysbiosis and neuroinflammation in perioperative neurocognitive disorders, including POD, and highlighted the gut-brain axis as a potential target for therapeutic intervention (24). However, most current evidence remains indirect, and further clinical studies are required to establish causality and translational relevance.

## 3 Postoperative delirium: epidemiology and clinical features

### 3.1 Definition and diagnostic criteria

Postoperative delirium is an acute and fluctuating neuropsychiatric syndrome occurring in the postoperative period. According to DSM-5, the diagnostic criteria include: (1) a disturbance in attention and awareness; (2) an acute onset with a fluctuating course; and (3) an additional cognitive disturbance (e.g., memory impairment, disorientation, or perceptual disturbance) (25, 26).

The Confusion Assessment Method (CAM) is the most widely used bedside screening tool for delirium. A diagnosis based on CAM requires the presence of four features: (1) acute onset and fluctuating course, (2) inattention, (3) disorganized thinking, and (4) altered level of consciousness. Delirium is diagnosed when features (1) and (2) are present together with either (3) or (4). Initial validation studies reported a sensitivity of 94%–100% and specificity of 90%–95% (27). Subsequent systematic review and meta-analysis have further supported the strong diagnostic performance of CAM-ICU and the Intensive Care Delirium Screening Checklist (ICDSC) in critically ill populations (28).

For the assessment of delirium severity, the Memorial Delirium Assessment Scale (MDAS) evaluates domains including consciousness, attention, cognition, psychomotor activity, and sleep–wake cycle disturbances across 10 items (each scored from 0 to 3, with a total score ranging from 0 to 30). Although no universally accepted cutoff exists, a score ≥10 is generally considered indicative of clinically significant delirium, while some postoperative studies have adopted a lower threshold (e.g., ≥5) for screening purposes (29).

### 3.2 Risk factors and prognosis

The development of POD is multifactorial and likely reflects a complex interplay between patient-related vulnerabilities, surgical factors, and perioperative management (1). Non-modifiable risk factors include advanced age, male sex, pre-existing cognitive impairment, and a high burden of comorbidities (e.g., cardiovascular disease, diabetes mellitus, hepatic or renal dysfunction). Modifiable risk factors include preoperative exposure to anticholinergic medications, intraoperative hemodynamic instability, excessive depth of anesthesia, poorly controlled postoperative pain, sleep disruption, and the use of physical restraints (30).

Postoperative delirium is consistently associated with adverse clinical outcomes. Evidence from cohort studies and meta-analyses indicates that POD is linked to prolonged hospital stay, increased post-discharge mortality, higher readmission rates, and impaired long-term functional recovery (31, 32). Furthermore, some patients do not regain their preoperative cognitive baseline, suggesting that POD may contribute to or accelerate underlying neurodegenerative processes (33).

### 3.3 Specificity of postoperative delirium in patients with liver disease

#### 3.3.1 Background of liver disease

Liver disease comprises a broad spectrum of conditions, ranging from acute hepatitis to chronic disorders, including cirrhosis of various etiologies (e.g., MASLD, alcohol-related liver disease, viral hepatitis) (9, 10). Disease severity is commonly assessed using the Child–Pugh classification (Classes A, B, and C) and the MELD score. These systems incorporate key clinical and laboratory parameters—including serum bilirubin, albumin, prothrombin time, and renal function—to estimate hepatic reserve and overall prognosis (34). Advanced liver disease is frequently associated with complications such as portal hypertension, ascites, variceal bleeding, HE, and SBP (35).

Surgical indications in this population are heterogeneous. Elective procedures may be considered in patients with well-compensated cirrhosis (Child–Pugh Class A or carefully selected Class B) following appropriate preoperative optimization. In contrast, emergency surgery (e.g., for trauma, gastrointestinal bleeding, or acute abdomen) is often unavoidable regardless of hepatic function (35). Importantly, the presence of cirrhosis-even at a compensated stage-is associated with a significantly increased risk of perioperative morbidity and mortality, highlighting the importance of careful risk stratification and multidisciplinary perioperative management (36).

#### 3.3.2 High risk of POD in patients with liver disease

Patients with liver disease represent a particularly high-risk population for the development of POD. The incidence of POD is significantly increased in cirrhotic patients undergoing both hepatic and non-hepatic surgeries and appears to correlate with worsening Child–Pugh class (34). Similarly, liver transplant recipients exhibit a high incidence of POD, which may negatively affect postoperative recovery and long-term graft and patient outcomes (37).

Multiple interrelated mechanisms likely contribute to this increased susceptibility. First, baseline neurocognitive vulnerability is common in liver disease, ranging from minimal HE-characterized by subtle cognitive impairment detectable only through specialized testing—to overt encephalopathy. These conditions may lower the threshold for perioperative neurocognitive deterioration (35). Second, impaired hepatic detoxification leads to the accumulation of gut-derived neurotoxic substances, such as ammonia and manganese, which can adversely affect neuronal function (38). Third, chronic systemic inflammation is frequently present, driven by endotoxemia and bacterial translocation resulting from intestinal barrier dysfunction, a hallmark of advanced liver disease (8, 39).

In addition, altered hepatic metabolism may modify the pharmacokinetics and pharmacodynamics of sedatives, analgesics, and anesthetic agents, increasing the risk of drug-related neurotoxicity (35). Malnutrition and sarcopenia, which are prevalent in chronic liver disease, may further reduce cognitive reserve and impair the physiological capacity to respond to surgical stress (35).

Collectively, these disturbances in neurocognitive function, inflammatory status, and metabolic capacity may render patients with liver disease particularly vulnerable to the additional stressors of anesthesia and surgery, thereby substantially increasing the risk of POD.

## 4 Gut microbiota and postoperative delirium: clinical evidence

It is important to acknowledge that the current clinical evidence linking gut microbiota to POD is predominantly associative. Although mechanistic insights have been derived from preclinical studies, causal relationships in human populations have not yet been definitively established.

Accordingly, the findings discussed below should be interpreted as hypothesis-generating. The proposed mechanisms represent biologically plausible pathways that require further validation through well-designed longitudinal studies and interventional clinical trials.

### 4.1 Overview of clinical studies

While animal studies have provided mechanistic support for the involvement of gut microbiota in perioperative neurocognitive regulation, clinical evidence in humans remains limited. Liu et al. (40) reported an association between preoperative gut microbial composition and the development of POD in patients undergoing gastric cancer surgery. Specifically, the delirium group demonstrated higher preoperative abundances of potentially pathogenic taxa, including Proteobacteria, Enterobacteriaceae, and *Escherichia*/*Klebsiella*, along with reduced levels of certain SCFA-producing genera. Although these findings suggest that baseline microbiota profiles may contribute to a risk phenotype for POD, the study design does not allow for conclusions regarding causality or temporal directionality.

Subsequent prospective cohort studies have expanded this evidence by incorporating postoperative microbial analyses (23). In one study of patients aged ≥65 years undergoing orthopedic or spine surgery, POD was assessed using the CAM alongside 16S rRNA gene sequencing. The results indicated that a higher relative abundance of Parabacteroides distasonis-a Gram-negative anaerobe within the phylum Bacteroidetes—was associated with an increased risk of POD (adjusted odds ratio [OR] = 2.13, 95% confidence interval [CI]: 1.09–4.17, *P* = 0.026) (23). In contrast, *Prevotella* and *Collinsella* showed non-significant decreasing trends in the delirium group.

Importantly, perioperative factors-including antibiotic exposure, bowel preparation, analgesic and sedative use, proton pump inhibitor therapy, and dietary changes-can substantially influence gut microbial composition. Therefore, future studies should incorporate rigorous control of these confounders and adopt longitudinal designs with repeated sampling to better clarify the temporal dynamics and potential causal relationships between gut microbiota and POD.

#### 4.1.1 Relevance to inflammatory bowel disease and shared pathways

Although the aforementioned study focused on orthopedic surgery patients, the observed association between Parabacteroides distasonis abundance and POD risk raises the question of whether inflammatory bowel disease (IBD)-a prototypical condition characterized by chronic intestinal inflammation and dysbiosis-shares overlapping mechanistic pathways with POD. In patients with IBD, *P. distasonis* has been reported to be enriched during active disease and associated with intestinal inflammation (41). While this organism has demonstrated beneficial metabolic effects in non-inflammatory settings (e.g., improvement of metabolic parameters via succinate and secondary bile acid production) (42), its role appears to be context-dependent and may involve pro-inflammatory signaling and impairment of barrier integrity under inflammatory conditions.

Inflammatory bowel disease and POD may converge through several shared pathophysiological mechanisms. Chronic systemic inflammation in IBD is associated with elevated circulating pro-inflammatory cytokines, which may prime microglia and increase CNS vulnerability to secondary insults. Surgical stress may act as an acute trigger, amplifying inflammatory cascades and promoting neuroinflammation and delirium. Furthermore, intestinal barrier dysfunction and bacterial translocation—hallmarks of IBD—may be exacerbated by anesthesia and surgery, allowing microbial products such as LPS to enter the circulation and activate central immune pathways via the gut-brain axis (23, 41).

Collectively, these overlapping mechanisms suggest that patients with chronic intestinal inflammation may possess an elevated baseline susceptibility to POD, particularly in the presence of additional perioperative stressors. However, direct clinical evidence remains limited, and further studies are needed to determine whether IBD-or specific inflammation-associated microbial signatures-independently increases the risk of POD in surgical populations.

### 4.2 Biological significance of key bacterial genera

#### 4.2.1 Parabacteroides distasonis

*P. distasonis* is a strictly anaerobic, Gram-negative rod. Variations in its abundance have been associated with intestinal inflammation in patients with IBD (41). At the same time, this species has been implicated in host metabolic regulation through the production of bioactive metabolites, including succinate and secondary bile acids (42).

In the context of POD, it has been hypothesized that *P. distasonis* may contribute to increased risk through several potential mechanisms, including the promotion of intestinal inflammation and barrier dysfunction, modulation of neuroactive metabolite profiles, and competitive interactions with SCFA-producing commensal bacteria (40).

However, these proposed mechanisms are primarily derived from associative observations and indirect evidence. Further validation through integrated multi-omics approaches and well-designed interventional studies is required to clarify its specific role in POD pathophysiology.

#### 4.2.2 Prevotella

*Prevotella* is a genus of Gram-negative, anaerobic bacteria within the phylum Bacteroidetes, commonly found in the human gut and oral cavity. These bacteria are capable of fermenting complex dietary fibers and mucins, leading to the production of SCFAs-primarily acetate and propionate-which contribute to the maintenance of intestinal barrier integrity and modulation of immune responses (43).

The abundance of *Prevotella* has been associated with various chronic diseases in a context-dependent manner. Reduced levels have been reported in conditions such as rheumatoid arthritis, IBD, metabolic syndrome, and type 2 diabetes mellitus (43, 44). In these settings, decreased *Prevotella* abundance is often accompanied by reduced SCFA production and impaired immune regulation, potentially promoting a pro-inflammatory state. Conversely, higher abundance is typically observed in individuals consuming plant-rich, high-fiber diets and has been linked to improved metabolic profiles in certain populations (43).

The immunomodulatory effects of *Prevotella* are thought to be mediated, at least in part, by its metabolic products. SCFAs can promote regulatory T cell differentiation, enhance epithelial barrier function, and suppress excessive inflammatory responses (43). Therefore, a reduction in *Prevotella*—as suggested by the decreasing trend observed in patients with POD (23)—may hypothetically contribute to impaired immune homeostasis and barrier dysfunction, thereby facilitating a pro-inflammatory environment that increases susceptibility to perioperative neuroinflammation and delirium.

However, these interpretations remain speculative, and further studies in larger, well-characterized cohorts are required to confirm the role of *Prevotella* in POD.

#### 4.2.3 Collinsella

*Collinsella* is a genus of Gram-positive, anaerobic bacteria within the phylum Actinobacteria. Members of this genus are involved in carbohydrate fermentation and can produce SCFAs, as well as participate in bile acid metabolism (44).

However, emerging evidence indicates that the effects of *Collinsella* on host physiology are highly context-dependent. While some studies suggest a role in maintaining metabolic homeostasis, others have associated increased *Collinsella* abundance with pro-inflammatory states, altered gut permeability, and metabolic disturbances across different disease conditions.

These dual and context-specific roles underscore the complexity of interpreting *Collinsella* dynamics in clinical settings. Its potential involvement in POD remains unclear and warrants further investigation, particularly through studies integrating microbial composition with functional and metabolomic analyses.

##### 4.2.3.1 Association with chronic inflammatory and neurocognitive disorders

Several studies have identified *Collinsella* as being enriched in conditions characterized by chronic inflammation and metabolic dysfunction. In patients with rheumatoid arthritis, *Collinsella* abundance has been shown to correlate positively with disease activity and inflammatory markers (44). Similarly, in metabolic syndrome and non-alcoholic fatty liver disease (NAFLD), increased *Collinsella* levels have been associated with elevated endotoxemia and systemic inflammation (45).

Emerging evidence also implicates *Collinsella* in gut-brain axis dysregulation and neurocognitive disorders. Giner Pérez et al. (45) reported that *Collinsella* abundance was significantly altered in patients with cirrhosis-associated mild cognitive impairment, suggesting a potential link between systemic inflammation and neuroinflammation. Additionally, alterations in *Collinsella* have been observed in neurodegenerative diseases such as Parkinson’s disease and Alzheimer’s disease, although findings regarding the direction of these changes remain inconsistent across studies (21).

The mechanisms underlying these associations may involve several interconnected pathways. First, *Collinsella* may promote intestinal inflammation and impair epithelial barrier integrity through its structural components and metabolic products. Second, its involvement in bile acid metabolism may influence farnesoid X receptor (FXR) signaling, a pathway implicated in both hepatic and central nervous system inflammation. Third, increased *Collinsella* abundance has been associated with elevated circulating pro-inflammatory cytokines, which may contribute to microglial priming and the development of neuroinflammation (44, 45).

##### 4.2.3.2 Relevance to postoperative delirium

In the context of POD, Zhang et al. (23) reported a non-significant decreasing trend in *Collinsella* abundance among patients who developed delirium. Although this observation appears to contrast with findings of *Collinsella* enrichment in certain chronic inflammatory conditions, several explanations may account for this discrepancy.

First, the relationship between *Collinsella* and inflammation may be non-linear or context-dependent, with distinct roles in acute versus chronic settings. Second, the observed decrease may reflect a broader disruption of microbial communities involved in SCFA production, as butyrate and other SCFAs are known to exert anti-inflammatory and neuroprotective effects. Third, taxonomic analyses at the genus level may obscure important species- or strain-specific functional differences.

Taken together, while *Collinsella* is increasingly recognized as a relevant genus in gut-brain axis research, its role in POD remains incompletely understood. Further studies integrating metagenomic, metabolomic, and longitudinal clinical data are required to clarify its functional significance and establish potential causal relationships.

### 4.3 From association to causation: evidence gaps

Current evidence largely supports an association between gut microbiota composition and POD, rather than a definitive causal relationship. Establishing causality requires the fulfillment of several key criteria, including temporal precedence (i.e., microbial alterations occurring prior to POD onset), the presence of dose–response relationships, identification of coherent and verifiable biological mechanisms, and experimental validation demonstrating that modulation of the microbiota influences POD risk or severity (46). To date, few of these criteria have been adequately addressed in human studies, highlighting the need for more rigorous research.

Several limitations characterize the existing literature. Many studies rely on single postoperative timepoint sampling, which limits insight into dynamic microbiota changes across the perioperative period. Sample sizes are often small, with insufficient stratification and adjustment for confounding variables. Additionally, the widespread use of 16S rRNA gene sequencing restricts functional interpretation, as it provides limited resolution regarding microbial genes and metabolic activity. Furthermore, a lack of multicenter validation reduces the generalizability of current findings.

Future research should prioritize longitudinal study designs with serial sampling spanning the preoperative to postoperative phases. Integration of multi-omics approaches-such as shotgun metagenomics and metabolomics—will be essential to elucidate functional pathways. In addition, the application of causal inference frameworks and interventional strategies, including microbiota-modulating approaches such as fecal microbiota transplantation (FMT), may help establish a more robust causal link between gut dysbiosis and the pathogenesis of POD.

## 5 Liver disease, gut microecology, and perioperative neurocognitive risk

### 5.1 Gut dysbiosis in liver disease

Microbial alterations in patients with liver disease vary according to disease stage and underlying etiology. Bajaj et al. introduced the concept of the “cirrhosis dysbiosis ratio” to describe the imbalance between beneficial autochthonous taxa and potentially pathogenic bacteria (47). As cirrhosis progresses, this dysbiosis becomes more pronounced: the relative abundance of SCFA-producing bacteria decreases, while ammonia-producing and potentially pathogenic taxa expand. These compositional changes are accompanied by intestinal barrier dysfunction and an increased likelihood of bacterial translocation, collectively enhancing systemic exposure to inflammatory mediators and neurotoxic metabolites (39, 47).

Microbial signatures may also differ depending on the etiology of liver disease (48). In alcohol-related liver disease, microbial alterations are often associated with pathways involving ethanol metabolism and oxidative stress. MASLD is typically linked to disruptions in choline metabolism and increased trimethylamine production. In contrast, cirrhosis due to viral hepatitis may initially present with relatively modest microbial disturbances, although progressive dysbiosis develops with advancing hepatic dysfunction.

Overall, these findings suggest that patients with liver disease exhibit baseline abnormalities across the “microecology-barrier-metabolism” axis. Such pre-existing disturbances may lower the threshold for systemic inflammation and neurotoxicity, thereby increasing vulnerability to perioperative neurocognitive complications, including POD.

### 5.2 Intestinal barrier dysfunction and bacterial translocation: inflammatory amplifier

Intestinal barrier dysfunction and bacterial translocation associated with liver disease can promote endotoxemia and trigger systemic inflammatory cascades, thereby compromising BBB integrity and disrupting central immune homeostasis (8, 39). In cirrhosis, impairment of gut immunity involves both epithelial and gut vascular barriers, facilitating the translocation of microbial antigens into the portal circulation and potentially exacerbating hepatic injury (39).

Perioperative factors—including preoperative fasting, mechanical bowel preparation, antibiotic exposure, anesthesia-induced suppression of intestinal motility, and surgery-related catabolic stress—may further weaken intestinal barrier integrity. These insults can promote the translocation of bacterial components into the systemic circulation, amplifying inflammatory signaling.

Collectively, this process may function as an “inflammatory amplifier,” whereby perioperative stressors intensify pre-existing gut-derived inflammatory activity. This amplification not only increases hepatic metabolic burden but may also enhance neuroinflammatory responses, thereby contributing to the development of postoperative delirium.

### 5.3 Hepatic encephalopathy: neurotoxicity from gut to brain

Hepatic encephalopathy is a neuropsychiatric syndrome arising from hepatic insufficiency, in which gut-derived neurotoxins play a central pathogenic role (38). Ammonia represents the most critical of these neurotoxins. Intestinal bacteria-particularly urease-positive organisms such as *Klebsiella* and *Proteus*-generate ammonia through the degradation of proteins and urea, which subsequently enters the portal circulation. In the setting of liver failure, impaired hepatic clearance allows ammonia to accumulate in the bloodstream and access the CNS. Within the CNS, ammonia is converted to glutamine by astrocytic glutamine synthetase, increasing intracellular osmolarity, inducing astrocyte swelling, and ultimately leading to astrocytic dysfunction.

In addition to ammonia, the gut microbiota may produce several other neurotoxic compounds (38):

Manganese-accumulates in the basal ganglia and may contribute to Parkinsonism-like manifestations.

Indole compounds-can interact with benzodiazepine receptors and enhance GABAergic inhibitory neurotransmission.

Endotoxins-activate microglial TLR4 signaling, promoting neuroinflammation.

**FIGURE 1:**
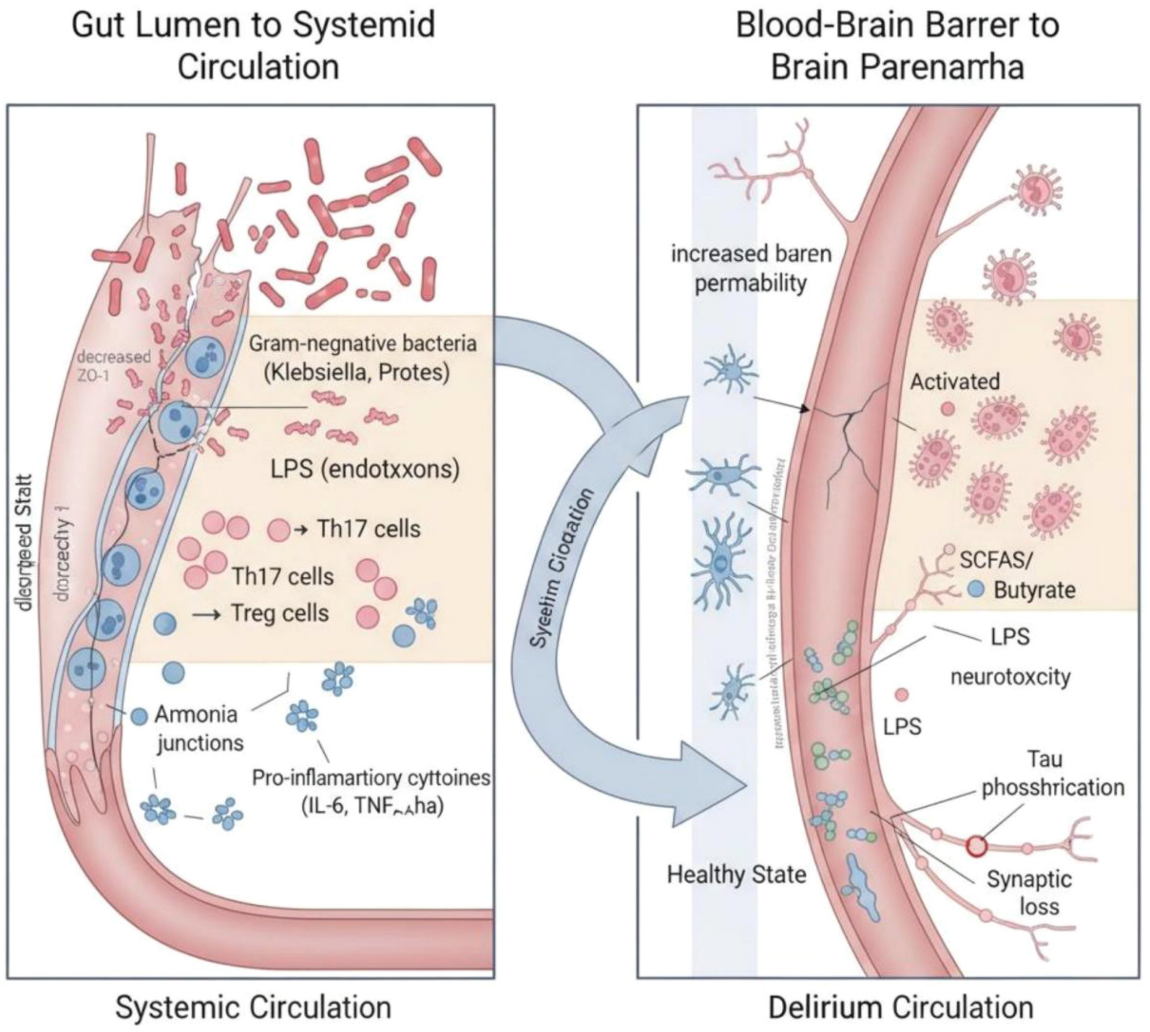
Cellular and molecular mechanisms connecting gut dysbiosis to neuroinflammation. Schematic overview of gut dysbiosis-driven neuroinflammation in the pathogenesis of delirium. Within the intestinal lumen, dysbiosis is characterized by an overgrowth of Gram-negative bacteria such as *Klebsiella* and *Proteus*, which elaborate lipopolysaccharide (LPS) and ammonia (NH3). These microbial products, together with alterations in T-cell subset composition—specifically, an expansion of Th17 cells and a reduction in regulatory T cells (Tregs)—foster a pro-inflammatory milieu. LPS, ammonia, and pro-inflammatory cytokines (e.g., IL-6, TNF-α) gain access to the systemic circulation. At the level of the blood–brain barrier (BBB), these circulating factors promote increased BBB permeability and trigger microglial activation, thereby driving neuroinflammation and ultimately contributing to the pathophysiology of delirium.

Figure 1 illustrates the cellular and molecular mechanisms linking gut dysbiosis to neuroinflammation. Dysbiosis is characterized by the overgrowth of Gram-negative bacteria such as *Klebsiella* and *Proteus*, leading to increased production of LPS and ammonia. These microbial products, together with alterations in T-cell subsets—namely, expansion of Th17 cells and reduction of Tregs-contribute to a pro-inflammatory milieu. LPS, ammonia, and pro-inflammatory cytokines (e.g., IL-6, TNF-α) enter the systemic circulation and act on the BBB, increasing its permeability and promoting microglial activation. This cascade ultimately drives neuroinflammation and contributes to the pathophysiology of delirium.

The synergistic effects of these neurotoxins likely contribute to the marked clinical heterogeneity observed in patients with HE. Anesthesia and surgery may further exacerbate susceptibility to HE (35). Preoperative fasting and mechanical bowel preparation can disrupt gut microbial composition, while anesthetic agents suppress intestinal motility and promote bacterial overgrowth. In addition, surgical stress enhances catabolic activity, leading to increased endogenous ammonia production. The perioperative use of sedatives and analgesics may further complicate the clinical picture by either masking or precipitating disturbances in consciousness.

As a result, in patients with underlying liver disease, postoperative alterations in mental status present a significant diagnostic challenge. Differentiating between HE exacerbation, drug-induced encephalopathy, and POD is often difficult, requiring careful clinical assessment and consideration of overlapping pathophysiological mechanisms.

### 5.4 Gut-liver-anesthesia axis: an integrative conceptual framework

Building upon these mechanistic insights, Shi et al. proposed the concept of the “gut-liver-anesthesia axis,” which integrates gut microecological disturbances, hepatic vulnerability, and anesthetic drug exposure into a unified perioperative risk model (11). The core logic of this framework may be summarized as follows:

(1). First hit: Patients with liver disease may harbor pre-existing gut dysbiosis and intrinsic intestinal barrier vulnerability.
(2). Second hit: Anesthetic agents and surgical stress may further disrupt gut microecology and compromise barrier integrity.
(3). Inflammatory amplification: Barrier dysfunction may facilitate the translocation of bacteria and microbial toxins, thereby triggering systemic inflammation and exacerbating both hepatic injury and neuroinflammation.
(4). Bidirectional feedback: The gut microbiota may modulate hepatic drug-metabolizing enzyme activity, thereby influencing anesthetic pharmacokinetics and drug sensitivity, potentially establishing a bidirectional feedback loop (49).

The present review extends the “gut-liver-anesthesia axis” framework in two key respects. First, it focuses specifically on patients with pre-existing liver disease, a population in whom baseline disturbances in microecology, barrier function, and immune homeostasis may confer heightened susceptibility to additive perioperative insults, rendering this framework particularly clinically relevant. Second, it incorporates a detailed analysis of how individual anesthetic agents may differentially affect gut microbiota composition and intestinal barrier integrity (summarized in Table 1), along with an examination of bidirectional pharmacomicrobiomic interactions mediated by nuclear receptors-particularly PXR and FXR-that link microbial composition to hepatic drug metabolism.

**TABLE 1:** Comparative effects of anesthetic agents on gut microbiota composition and intestinal barrier function.

Anesthetic agent	Reported or proposed effects on gut microbiota	Reported or proposed effects on intestinal barrier function	Potential implications in liver disease	References
Sevoflurane	Animal studies show sevoflurane reduces gut microbial α-diversity, depletes Firmicutes/Clostridiales, and expands Proteobacteria; dysbiosis persists for ≥14 days.	Sevoflurane may impair intestinal tight junction integrity and increase permeability; mechanisms involve brain-gut axis signaling.	May aggravate bacterial translocation, systemic inflammation, and neurocognitive vulnerability in liver disease.	(50, 63, 64)
Opioids	Chronic opioid exposure induces dysbiosis with expansion of Gram-positive opportunistic pathogens and depletion of beneficial taxa (e.g., *Lachnospiraceae*).	Opioids disrupt tight junctions via TLR4-dependent mechanism; intestinal stasis promotes SIBO; reduces antimicrobial activity of ileal epithelium.	Particularly relevant in cirrhosis: worsens constipation, bacterial translocation, SBP risk, and HE. Opioid-sparing strategies preferred.	(35, 57, 58)
Propofol	Clinical study shows propofol may lead to lesser changes in intestinal microbes associated with neurological diseases compared to sevoflurane.	Propofol protects intestinal barrier by downregulating miR-155 and inhibiting TLR4/NF-κB pathway, promoting tight junction protein (occludin, ZO-1) expression; increases *Lactobacillus* on mucosal surface.	Propofol-based TIVA may be favorable in liver disease; direct evidence in cirrhotic patients is lacking.	(65, 66)
Remimazolam	Direct evidence on gut microbiota currently lacking; general anesthetics may alter gut microbiota by affecting barrier function, bacterial proliferation, or bile acid metabolism.	Animal study shows remimazolam protects intestinal barrier in sepsis by mitigating mitochondrial oxidative damage; improves survival from 12.5% to 68.75%; restores villus architecture; increases ZO-1 expression.	Organ-independent metabolism offers pharmacokinetic advantages in cirrhosis; microbiota implications require further study.	(49, 67, 68)
Lidocaine (IV)	Animal study suggests lidocaine attenuates POD by reshaping intestinal flora (*Prevotellaceae*, *Rikenellaceae*, *Faecalibaculum* as potential markers).	Lidocaine promotes tight junction proteins (ZO-1, occludin, claudin-5), reduces BBB permeability, and restores intestinal barrier. Spinal anesthesia preserves claudin-1, occludin, and mucin production.	Opioid-sparing benefits; may indirectly reduce gut dysfunction in liver disease; further studies needed.	(69, 70)
Sugammadex	No direct evidence for clinically meaningful effects on gut microbiota; as a γ-cyclodextrin, theoretical fermentation potential is hypothetical.	No established direct adverse effect on intestinal barrier reported; oral bioavailability < 4% due to enzymatic degradation.	Main relevance is pharmacologic (rapid reversal of neuromuscular blockade); no specific gut-microecology concerns in liver disease identified.	(71)

ZO-1, zonula occludens-1; SIBO, small intestinal bacterial overgrowth; TLR4, toll-like receptor 4; TIVA, total intravenous anesthesia; HE, hepatic encephalopathy; SBP, spontaneous bacterial peritonitis; POD, postoperative delirium. Evidence summarized includes preclinical animal studies and limited clinical data.

These refinements provide a more granular, mechanism-based framework for understanding POD risk and for guiding targeted perioperative strategies in this high-risk population.

The clinical implications of this framework are multifaceted. First, gut microecology may serve not only as a risk stratification marker but also as a modifiable therapeutic target. Second, anesthetic management should consider potential effects on intestinal microecology and hepatic metabolic capacity. Third, interventions targeting the gut-liver axis may simultaneously mitigate hepatic complications and reduce perioperative neurocognitive risk.

**FIGURE 2:**
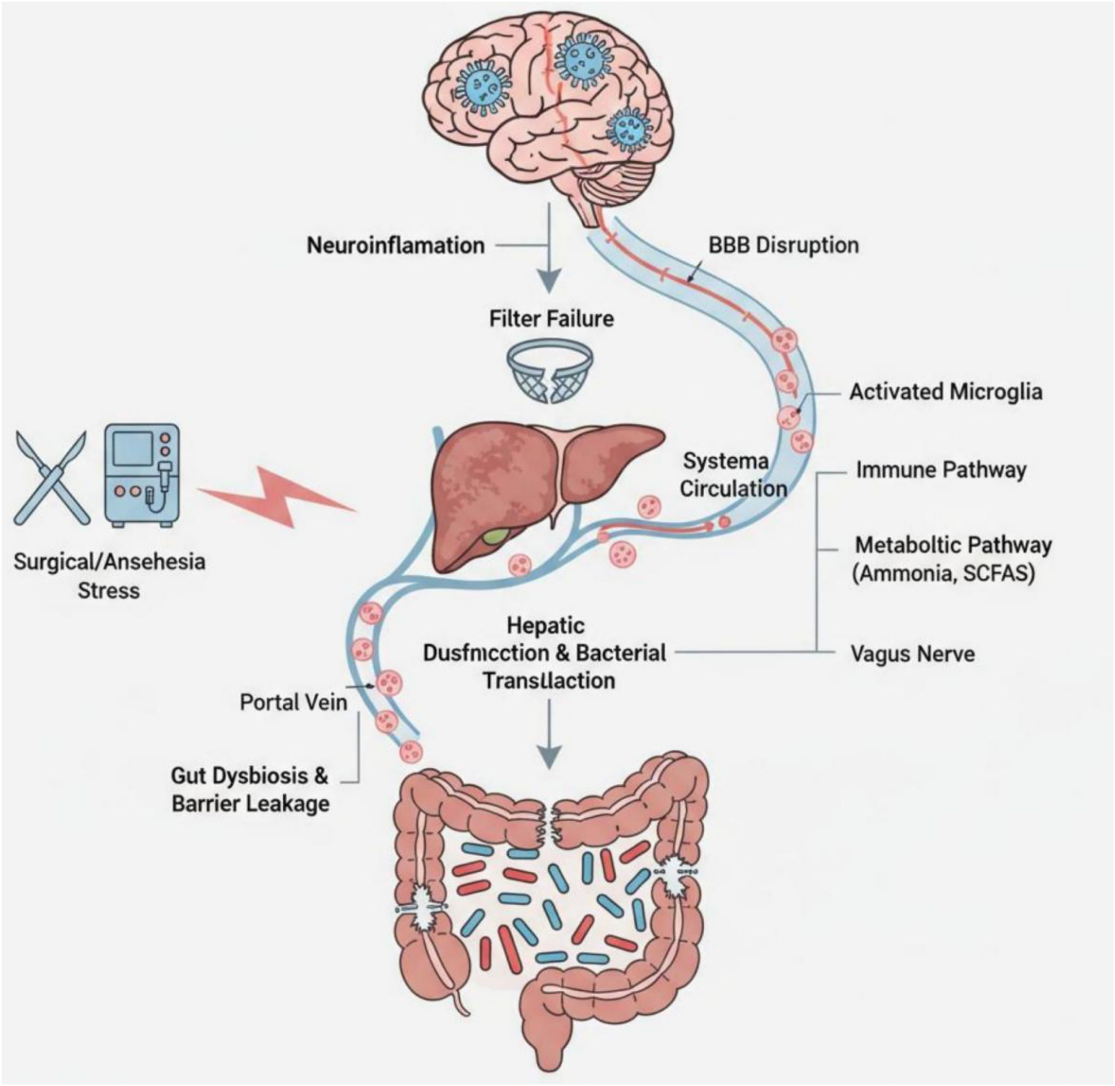
The concept of the gut-liver-anesthesia axis in POD. Schematic illustration of the gut–liver–brain axis in the pathogenesis of postoperative delirium. Gut dysbiosis and impaired intestinal barrier integrity permit bacterial products and toxins to enter the portal circulation and reach the liver. Hepatic dysfunction compromises the clearance of these compounds and facilitates bacterial translocation into the systemic circulation. Surgical stress and exposure to anesthetic agents (denoted as “Surgical/Anesthesia Stress”) further exacerbate gut barrier disruption and amplify systemic inflammation. Microbial metabolites (e.g., ammonia, short-chain fatty acids) and immune mediators disseminate via the systemic circulation, where they engage immune and metabolic signaling pathways. The vagus nerve provides a direct neural conduit for communication between the gut and the central nervous system. Collectively, systemic inflammation and microbial-derived signals disrupt blood–brain barrier integrity, activate microglia, and provoke neuroinflammation, thereby contributing to the development of postoperative delirium.

Figure 2 presents a schematic representation of this multidimensional interaction model, illustrating the progression from gut dysbiosis to impaired gut-liver barrier function and amplified systemic inflammation, ultimately leading to adverse CNS outcomes under the added stress of surgery.

## 6 Impact of anesthetic agents on gut microecology

### 6.1 Inhalational anesthetics

Animal studies suggest that sevoflurane exposure can rapidly reduce gut microbial α-diversity, with depletion of Firmicutes and Clostridiales and a concurrent expansion of Proteobacteria (50). This compositional shift resembles antibiotic-induced dysbiosis, implying that inhalational anesthetics may exert antibacterial-like effects on the gut ecosystem.

Beyond direct effects on microbial composition, inhalational agents may also induce cytotoxic effects on intestinal epithelial cells. In neonatal mice, repeated exposure to sevoflurane has been shown to damage the intestinal mucus layer, downregulate tight junction proteins such as occludin and ZO-1, and increase intestinal barrier permeability (51).

Importantly, sevoflurane-induced dysbiosis may influence cognitive function through the gut-brain axis. Fecal microbiota transplantation from sevoflurane-exposed mice into germ-free recipients has been shown to transfer memory deficits, indicating that the neurocognitive effects of sevoflurane may be partially mediated by alterations in gut microbiota composition (52).

More recent evidence further suggests that sevoflurane-induced dysbiosis contributes to cognitive impairment in aged mice via activation of the NLRP3 inflammasome. Notably, probiotic intervention has been reported to mitigate these effects, potentially through restoration of microbial balance and attenuation of inflammatory signaling (53).

### 6.2 Intravenous anesthetics

#### 6.2.1 Propofol

Propofol, a cornerstone agent for total intravenous anesthesia, appears to exert a comparatively modest impact on gut microecology. Wilson and Nicholson (54) reported that although intestinal motility is suppressed during propofol infusion, gastrointestinal function tends to recover more rapidly following discontinuation compared with inhalational anesthesia.

The lipid emulsion formulation of propofol may theoretically provide a substrate that supports bacterial growth; however, propofol itself exhibits intrinsic anti-inflammatory properties. Notably, it can inhibit TLR4-mediated endotoxin signaling, which may partially mitigate the systemic inflammatory effects associated with bacterial translocation (55).

Despite these potentially protective properties, high-dose or prolonged propofol administration has been associated with reductions in the abundance of genera such as *Prevotella* and *Lactobacillus*, indicating that sustained exposure may still induce measurable alterations in gut microbial composition (55).

#### 6.2.2 Ketamine

Ketamine, an NMDA receptor antagonist, may influence the gut-brain axis through distinct mechanisms. De Kock et al. (56) demonstrated that ketamine exerts anti-inflammatory effects in experimental sepsis models via inhibition of NF-κB activation.

In addition, the sympathomimetic properties of ketamine may help preserve splanchnic perfusion pressure, potentially providing protective effects on intestinal barrier integrity, particularly in cirrhotic patients who are prone to hemodynamic instability.

Emerging evidence further suggests that ketamine may exert a relatively limited impact on postoperative gut microbiota composition in aged mice compared with sevoflurane, indicating potential advantages in high-risk elderly populations (23).

#### 6.2.3 Remimazolam

Remimazolam is a novel ultra-short-acting benzodiazepine, and its specific effects on gut microecology remain incompletely characterized. High-quality sequencing data delineating its direct impact on microbial composition are currently limited.

### 6.3 Opioids

Opioids are among the most disruptive classes of perioperative medications with respect to gut microecology, exerting deleterious effects through multiple intersecting mechanisms (57). A primary mechanism involves μ-opioid receptor-mediated inhibition of intestinal motility within the enteric nervous system, leading to opioid-induced bowel dysfunction characterized by constipation, bloating, and intestinal stasis. Such stasis may promote SIBO, a condition already prevalent in cirrhotic patients, which opioid exposure may further exacerbate, thereby increasing the bacterial burden available for translocation.

More critically, opioids may directly impair intestinal barrier integrity. Velasco et al. (35) demonstrated that morphine disrupts tight junction architecture via a TLR4-dependent mechanism, increasing intestinal permeability even in the absence of overt stasis. Thomas et al. (58) further reported that both acute and chronic opioid exposure are associated with a characteristic dysbiosis, marked by expansion of Gram-positive opportunistic pathogens (e.g., *Staphylococcus*, *Enterococcus*) and depletion of beneficial autochthonous taxa (e.g., Lachnospiraceae). Notably, Enterococcus species are frequently implicated in SBP and bacteremia in cirrhotic individuals, rendering opioid-induced enterococcal expansion particularly concerning in this population.

Dysbiosis and barrier disruption may collectively establish a vicious cycle: systemic inflammation can promote opioid tolerance, necessitating escalating doses to achieve adequate analgesia, while higher opioid exposure may in turn further compromise gut integrity (59). This cycle may help explain why postoperative pain management in patients with liver disease often presents a clinical paradox, characterized by increased opioid requirements alongside reduced analgesic efficacy.

### 6.4 Regional anesthesia and other agents

Compared with systemic anesthetic agents, regional anesthesia techniques may exert protective effects on gut microecology by reducing exposure to potentially enterotoxic drugs. Epidural anesthesia blocks sympathetic innervation to the viscera, thereby improving intestinal mucosal perfusion and mitigating the risk of ischemia-reperfusion injury during major surgical procedures (60). By providing effective analgesia, regional techniques may also reduce systemic requirements for opioids and inhalational anesthetics, thereby indirectly limiting their adverse effects on gut microbiota (61).

Intravenous lidocaine has attracted increasing attention in perioperative care. Systematic reviews indicate that intravenous lidocaine possesses anti-inflammatory and analgesic properties while preserving gastrointestinal function, facilitating earlier recovery of bowel activity and reducing the incidence of postoperative ileus (62).

Overall, anesthetic agents differ substantially in their effects on gut microbiota composition and intestinal barrier integrity. As summarized in Table 1, opioids and sevoflurane appear to induce the most pronounced disruptions in microbial diversity and barrier function, whereas lidocaine and regional anesthesia are associated with a comparatively lower risk profile.

## 7 Bidirectional regulation between gut microbiota and anesthetic drug metabolism

### 7.1 The concept of pharmacomicrobiomics

Pharmacomicrobiomics is an interdisciplinary field that examines the interactions between gut microbiota and drug metabolism, with particular emphasis on how microbial genetic and enzymatic profiles influence drug efficacy and toxicity (72). The metabolic capacity of the gut microbiota exceeds that of the human genome and includes a wide range of enzymes such as β-glucuronidases, sulfatases, azoreductases, and nitroreductases. These enzymes can modify drugs prior to systemic absorption or reactivate metabolites that have been excreted into the intestinal lumen via bile.

Morphine provides a representative example of this interaction. Hepatic metabolism converts morphine into morphine-3-glucuronide, which is largely inactive, and morphine-6-glucuronide, which retains potent analgesic activity. Gut bacterial β-glucuronidases can hydrolyze these conjugates, releasing free morphine that may be reabsorbed into the systemic circulation, thereby establishing an enterohepatic recirculation loop (73).

**FIGURE 3:**
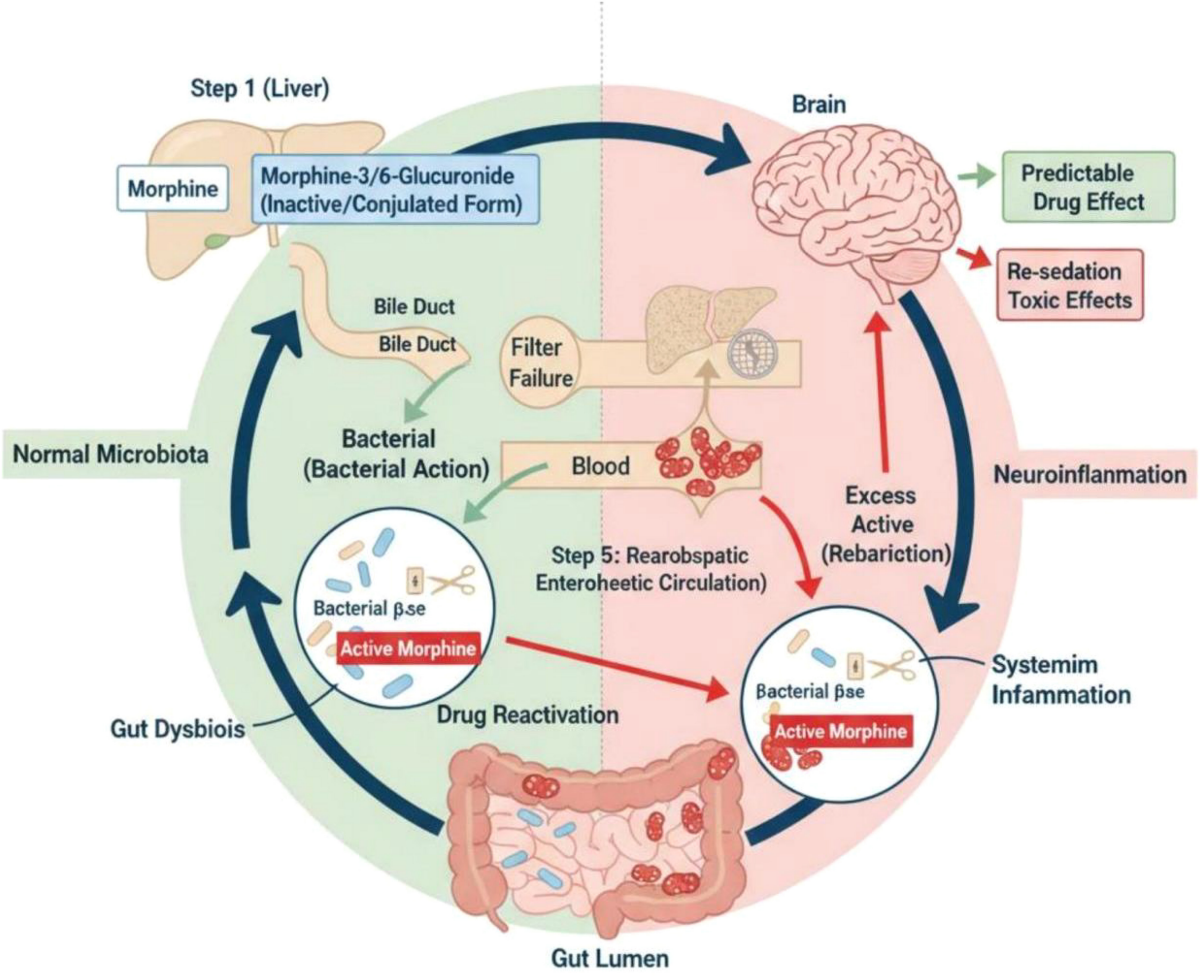
Pharmacomicrobiomics: the role of gut bacteria in anesthetic drug metabolism and recirculation. Schematic representation of morphine reactivation via gut microbial β-glucuronidase activity and enterohepatic circulation. Morphine undergoes hepatic glucuronidation to yield inactive morphine-3-glucuronide and active morphine-6-glucuronide (Step 1). These conjugates are excreted via the bile duct into the intestinal lumen. Within the gut, commensal bacteria expressing β-glucuronidase hydrolyze the glucuronide conjugates, liberating free active morphine (Step 5: reabsorption via enterohepatic circulation). Under conditions of a eubiotic microbiota, this process results in predictable drug effects. In the setting of gut dysbiosis, however, altered microbial β-glucuronidase activity may lead to excessive drug reactivation, thereby contributing to systemic inflammation, neuroinflammation, and adverse clinical outcomes such as re-sedation and toxicity.

Under conditions of dysbiosis, fluctuations in β-glucuronidase-producing bacterial populations may render this process highly variable. This variability may contribute to the phenomenon of delayed or recurrent sedation observed in some patients after surgery. Figure 3 illustrates the role of bacterial β-glucuronidase in drug deconjugation, reactivation, and enterohepatic circulation, highlighting a key mechanism through which gut microbiota can modulate drug effects.

### 7.2 Microbiota-mediated regulation of hepatic drug-metabolizing enzymes

Beyond the direct biotransformation of drugs by microbial enzymes, the gut microbiota may exert a more fundamental influence on drug disposition by regulating host drug-metabolizing enzyme systems, particularly the hepatic cytochrome P450 (CYP) family. This regulatory effect is largely mediated by microbial metabolites that function as ligands for nuclear receptors—a class of transcription factors that sense xenobiotics and endogenous signaling molecules and coordinate adaptive transcriptional responses (72).

#### 7.2.1 Nuclear receptors PXR and FXR as key regulators of drug metabolism

Pregnane X receptor (PXR) and constitutive androstane receptor (CAR) are key xenobiotic sensors that regulate the expression of phase I enzymes (e.g., CYP3A4, CYP2C), phase II enzymes (e.g., UDP-glucuronosyltransferases), and drug transporters such as P-glycoprotein. Upon activation by a broad spectrum of endogenous and exogenous ligands—including drugs, bile acids, and microbial metabolites—PXR translocates to the nucleus, heterodimerizes with retinoid X receptor (RXR), and binds to response elements within the promoter regions of target genes, thereby inducing their transcription (72, 74). This inducible regulatory system plays a critical role in enhancing the clearance of potentially toxic compounds, including commonly used perioperative agents such as midazolam, fentanyl, and other opioids.

Farnesoid X receptor (FXR) serves as the principal bile acid sensor within the enterohepatic circulation. Activated by primary bile acids and microbially derived secondary bile acids, FXR regulates bile acid synthesis, transport, and detoxification, thereby maintaining bile acid homeostasis and preventing toxic accumulation in the liver and intestine (63). Beyond its canonical role in bile acid metabolism, FXR may also influence the expression of CYP3A4 and other drug-metabolizing enzymes, thereby linking gut microbial activity and nutritional status to hepatic drug clearance capacity.

#### 7.2.2 Gut microbiota may modulate nuclear receptor activity via metabolite signaling

Importantly, the gut microbiota may regulate nuclear receptor activity through the production of endogenous ligands. Primary bile acids synthesized in the liver are secreted into the intestinal lumen, where gut bacteria—particularly members of Clostridium clusters XIVa and XI—convert them into secondary bile acids (e.g., deoxycholic acid, lithocholic acid) via deconjugation and 7α-dehydroxylation (63). These secondary bile acids act as potent ligands for both FXR and PXR. In patients with liver disease, dysbiosis often leads to reduced production of secondary bile acids, which may impair FXR and PXR activation and consequently alter the expression of CYP3A4 and other drug-metabolizing enzymes (74).

The clinical implications of this microbiota–nuclear receptor crosstalk are particularly relevant in the perioperative context. First, interindividual variability in gut microbiota composition may contribute to differences in anesthetic drug metabolism and sensitivity. Second, in patients with cirrhosis and concomitant dysbiosis, reduced secondary bile acid synthesis may weaken FXR and PXR signaling, potentially diminishing drug clearance capacity and increasing susceptibility to drug-induced encephalopathy (35). Third, anesthetic agents and perioperative interventions that disrupt gut microbiota—such as antibiotics and opioids—may secondarily influence hepatic drug metabolism, thereby establishing a bidirectional feedback loop with important implications for both drug efficacy and safety (49).

## 8 Microbiota-based perioperative intervention strategies

The gut dysbiosis, intestinal barrier dysfunction, and systemic inflammation characteristic of chronic liver disease provide a strong mechanistic rationale for microbiota-targeted perioperative interventions. As reviewed by Woodhouse et al. (75), strategies aimed at restoring beneficial commensal bacteria, suppressing pathogenic taxa, or protecting the intestinal ecosystem from perioperative disruption may help attenuate gut-liver-brain axis dysfunction and thereby reduce neurocognitive vulnerability in patients with cirrhosis. Recent reviews have further summarized the therapeutic potential of targeting the gut microbiome in chronic liver disease, including its possible relevance to perioperative neurocognitive outcomes (76).

Within this framework, the present section evaluates current evidence regarding probiotics and synbiotics, rifaximin, fecal microbiota transplantation (FMT), and perioperative supportive strategies that may help preserve gut homeostasis. Importantly, although several of these interventions have demonstrated benefits in improving liver-related outcomes or reducing postoperative infections, direct evidence supporting their efficacy in preventing postoperative delirium (POD) remains limited.

Therefore, the microbiota-based interventions discussed below should be regarded as investigational or adjunctive strategies, rather than established therapies for POD prevention.

### 8.1 Restoring commensal balance: probiotics and synbiotics

Probiotics and synbiotics are designed to restore beneficial microbial populations, strengthen epithelial barrier integrity, competitively inhibit pathogenic organisms, and modulate both mucosal and systemic immune responses. These effects are particularly relevant in chronic liver disease, where dysbiosis, increased intestinal permeability, and endotoxemia may contribute to neuroinflammation and heightened perioperative brain vulnerability.

In patients undergoing major liver surgery, a meta-analysis by Wu et al. (77) reported that perioperative synbiotics containing *Lactobacillus* and *Bifidobacterium* strains significantly reduced postoperative infection rates (RR = 0.42, 95% CI: 0.23–0.76) and shortened the duration of antibiotic therapy. Similarly, a systematic review by Kahn et al. (78) demonstrated lower infection rates in the probiotic/synbiotic group (18.5% vs. 31.8%, *P* < 0.001) and a reduction in hospital stay by approximately 3.2 days, with multi-strain formulations appearing more effective. A more recent meta-analysis (79) likewise confirmed a reduction in postoperative infectious complications (OR = 0.34, 95% CI: 0.25–0.45).

Nutritional optimization may further complement these interventions. Enteral nutrition is generally preferred over parenteral nutrition for preserving mucosal integrity and supporting gastrointestinal function (80). Soluble fiber can provide fermentable substrates for short-chain fatty acid production, while appropriate preoperative carbohydrate loading may help attenuate insulin resistance and support perioperative metabolic homeostasis.

From the perspective of postoperative delirium (POD), these strategies are of interest because they may reduce systemic inflammation, bacterial translocation, and gut-derived immune activation—processes implicated in delirium pathogenesis. However, no studies have directly evaluated probiotics or synbiotics for delirium prevention in patients with liver disease. In addition, heterogeneity in microbial strains, dosing regimens, treatment duration, and patient populations limits the generalizability of current evidence.

Accordingly, probiotics and synbiotics cannot currently be recommended for routine POD prevention. They should be regarded as promising investigational approaches, and well-designed randomized controlled trials with delirium-specific endpoints are needed to clarify their role.

### 8.2 Suppressing pathogenic taxa: rifaximin for hepatic encephalopathy vulnerability

Rifaximin is a minimally absorbed, gut-selective antibiotic with well-established efficacy in the management of hepatic encephalopathy (HE), making it one of the most clinically relevant microbiota-modulating therapies in cirrhosis. Its therapeutic effects extend beyond suppression of ammonia- and endotoxin-producing bacteria and may include modulation of microbial metabolic activity, attenuation of gut-derived inflammatory signaling, and partial restoration of intestinal barrier function (2, 3). Given that HE and postoperative delirium (POD) share overlapping pathophysiological mechanisms—including neuroinflammation, ammonia-related neurotoxicity, and increased cerebral vulnerability to systemic stress—rifaximin is of particular interest in the perioperative setting.

In the landmark randomized controlled trial by Bass et al. (81), rifaximin administered at 550 mg twice daily for six months significantly reduced the recurrence of HE compared with placebo (22.1% vs. 45.9%; HR = 0.42, 95% CI: 0.28–0.64, *P* < 0.001). More recently, a network meta-analysis by Fang et al. confirmed its benefits across multiple HE-related outcomes, including primary and secondary prevention, prevention of progression from minimal to overt HE, and improvement of minimal HE symptoms, although no significant mortality benefit was observed. These findings support rifaximin as the most well-established microbiota-targeted therapy for reducing HE-related neurocognitive vulnerability in cirrhotic patients.

However, its relevance to POD should be interpreted with caution. While perioperative continuation or optimization of rifaximin—often in combination with lactulose—may reduce the risk of HE exacerbation in high-risk cirrhotic patients, no clinical trials have directly demonstrated its efficacy in preventing POD. Accordingly, rifaximin should currently be regarded as an evidence-based strategy for HE prevention and stabilization, with potential but unproven benefits for reducing POD risk.

### 8.3 Reconstructing gut microecology: fecal microbiota transplantation

Fecal microbiota transplantation aims to restore microbial diversity and functional balance by transferring processed stool from healthy donors to recipients with severe dysbiosis. In liver disease—particularly cirrhosis and recurrent HE—this approach is of considerable interest, as it may improve microbial composition, enhance the production of beneficial metabolites such as short-chain fatty acids and secondary bile acids, and attenuate inflammatory and neurotoxic signaling along the gut–liver–brain axis. Consequently, FMT has emerged as a mechanistically compelling, though still experimental, therapeutic strategy in patients with microbiota-associated hepatic dysfunction.

In the phase II THEMATIC trial, Bajaj et al. (82) demonstrated that FMT was safe in patients receiving maximal HE therapy, with no FMT-related serious adverse events reported. A post hoc analysis suggested a lower recurrence of HE in FMT-treated patients compared with those receiving placebo (9% vs. 40%; OR = 0.15, 95% CI: 0.04–0.64), along with improvements in quality-of-life measures. Additional pilot studies have indicated potential benefits in severe alcoholic hepatitis (83) and metabolic dysfunction-associated steatotic liver disease (MASLD) (84, 85), although these findings remain preliminary and should be interpreted with caution.

Despite its strong mechanistic rationale, FMT has not been evaluated for the prevention of POD in the perioperative setting. Its use around the time of surgery also raises important safety and logistical concerns, including the risk of bacteremia, aspiration risk depending on the route of administration, challenges in protocol standardization, donor screening requirements, and uncertain applicability in immunocompromised patients or transplant candidates.

Accordingly, FMT should currently be regarded as an investigational intervention in this context and is not recommended as a routine strategy for POD prevention in patients with liver disease.

### 8.4 Protecting the gut from perioperative iatrogenic injury

In addition to direct microbiota-targeted therapies, perioperative supportive strategies may help preserve gut homeostasis and mitigate disruption of the gut-liver-brain axis. Surgical stress, fasting, antibiotic exposure, opioid administration, and hemodynamic instability can exacerbate dysbiosis, impair barrier integrity, and amplify systemic inflammation in patients with chronic liver disease. Accordingly, perioperative care that minimizes these insults may indirectly reduce vulnerability to neurocognitive complications.

Opioid-sparing approaches, including regional anesthesia techniques and selected multimodal analgesic regimens, may attenuate opioid-induced dysmotility, bacterial overgrowth, and constipation-related exacerbation of HE risk. Xu et al. (60) reported that opioid-sparing protocols may reduce postoperative ileus and infection-related complications. Thoracic epidural anesthesia has also been associated with improved splanchnic perfusion and attenuation of surgical stress responses, which may help preserve intestinal barrier function (86). By contrast, comparative evidence regarding the differential effects of TIVA versus volatile anesthesia on the gut microbiota remains limited and inconclusive (87).

Early postoperative enteral nutrition represents another key supportive measure, as it may help maintain mucosal integrity, promote intestinal motility, and reduce bacterial translocation; these principles are consistent with Enhanced Recovery After Surgery (ERAS) recommendations (88). Taken together, multimodal perioperative strategies integrating nutritional optimization, bowel function support, opioid minimization, and targeted prevention of HE provide a pragmatic framework for preserving gut homeostasis in vulnerable patients. However, direct evidence linking these bundled approaches to reduced POD incidence through microbiota-mediated pathways remains limited, and further prospective studies are warranted.

### 8.5 Evidence gaps and future directions

Although microbiota-based interventions have demonstrated benefits for liver-related outcomes—most notably rifaximin for HE and probiotics/synbiotics for postoperative infectious complications—direct evidence for POD prevention remains extremely limited. Several critical gaps merit emphasis.

First, most available studies are associative in nature or focus on liver-specific endpoints, and it remains unclear whether causal modulation of the gut microbiota can reduce POD risk. Second, no clinical trials have specifically evaluated these interventions for POD prevention, representing a fundamental evidence gap. Third, substantial heterogeneity exists across probiotic formulations, dosing regimens, FMT protocols, patient populations, and perioperative settings, thereby limiting comparability and generalizability. Fourth, safety considerations—particularly regarding FMT in perioperative or immunocompromised patients—require further dedicated investigation.

Future research should prioritize adequately powered, mechanism-informed randomized controlled trials that integrate longitudinal microbiome profiling, metabolomic analyses, and standardized delirium endpoints in high-risk liver disease populations. Such studies should directly assess whether preoperative microbiota optimization can reduce POD incidence. The principal microbiota-targeted strategies discussed above, along with their proposed mechanisms, supporting evidence, and relevance to POD, are summarized in Table 2.

**TABLE 2:** Microbiota-targeted strategies in patients with liver disease: proposed mechanisms, available evidence, and current recommendation for postoperative delirium prevention.

Strategy	Proposed mechanisms potentially relevant to POD	Evidence in liver disease (strength and focus)	Relevance to POD – interpretative note	References	Current recommendation for POD prevention (as of 2026)
Probiotics/synbiotics	Restore commensal balance (e.g., *Lactobacillus*, *Bifidobacterium*); strengthen intestinal barrier; reduce endotoxemia and systemic inflammation; may lower ammonia generation	Multiple meta-analyses of RCTs show reduced postoperative infections (OR ∼0.34–0.42) and shorter hospital stay in liver surgery; no direct evidence for delirium	Might indirectly affect POD susceptibility if gut-derived inflammation is attenuated, but this remains hypothetical; causal link not established	(77–79)	Not recommended for POD prevention outside of well-designed clinical trials
Rifaximin	Suppress ammonia- and endotoxin-producing bacteria; modulate microbial metabolic activity; reduce gut-derived inflammatory signaling	High-quality RCT evidence supports efficacy in preventing hepatic encephalopathy (HE) recurrence (HR ∼0.42) and improving minimal HE outcomes; no POD-specific trial	Most biologically plausible for patients with HE vulnerability, but direct extrapolation to POD remains unproven	(81)	Not recommended for POD prevention; use only as indicated for HE management
Fecal microbiota transplantation (FMT)	Restore overall microbial diversity and beneficial metabolites (SCFAs, secondary bile acids); suppress dysbiosis-associated pathogenic taxa	Small RCTs and pilot studies in cirrhosis/HE suggest safety and possible reduction of HE recurrence (*post hoc* analyses); no perioperative or POD-specific data	Mechanistically attractive for severe dysbiosis, but clinical applicability in the perioperative setting remains investigational; safety concerns (bacteremia, aspiration)	(82–84)	Investigational only – should not be used for POD prevention
Perioperative supportive strategies (opioid-sparing anesthesia, early enteral nutrition, bowel function support, HE prophylaxis)	Preserve gut motility and barrier function; reduce dysbiosis and bacterial translocation; attenuate systemic inflammation	Supported indirectly by Enhanced Recovery After Surgery (ERAS) and cirrhosis management literature; no direct microbiome-POD trials	A pragmatic, low-risk framework that may preserve gut homeostasis, but evidence remains indirect and hypothesis-generating	(60, 80, 88)	Reasonable supportive care based on general perioperative principles, but not validated specifically for POD prevention

POD, postoperative delirium; RCT, randomized controlled trial; HE, hepatic encephalopathy; SCFA, short-chain fatty acid; ERAS, Enhanced Recovery After Surgery; OR, odds ratio; HR, hazard ratio.

Evidence strength is based on the primary outcomes of the cited studies (e.g., infection, HE recurrence), not on POD prevention.

As of the publication date of this review, no microbiota-targeted intervention has been proven to reduce POD incidence in patients with liver disease. All strategies listed above should be considered experimental or supportive in the context of POD prevention unless and until confirmed by dedicated RCTs with delirium endpoints.

Reference numbers correspond to those in the reference list of the original manuscript.

## 9 Conclusion

Postoperative delirium in patients with liver disease likely arises from a multifactorial interplay involving gut microbiota dysbiosis, intestinal barrier dysfunction, bacterial translocation, hyperammonemia, systemic inflammation, and neuroinflammation along the gution alonrain axis. Current evidence supports a biologically plausible link between perioperative microbial perturbations and increased neurocognitive vulnerability, particularly in cirrhosis. However, this relationship is primarily derived from mechanistic insights and associative clinical observations, and should not be interpreted as causal.

Microbiota-targeted strategies-including probiotics, synbiotics, rifaximin, and FMT-have demonstrated benefits in selected liver-related outcomes, such as reduction of infectious complications or prevention of HE recurrence. These effects suggest potential upstream modulation of pathways relevant to POD susceptibility. Nevertheless, the clinical evidence remains indirect, as none of these interventions have been specifically evaluated for POD prevention in adequately designed prospective trials.

Among available approaches, rifaximin has the strongest evidence base in cirrhosis, particularly for HE-related outcomes, which may be relevant to perioperative cerebral vulnerability. In contrast, probiotics and synbiotics have shown benefits in postoperative recovery but lack evidence for neurocognitive endpoints. FMT remains mechanistically compelling but investigational, with unresolved safety and feasibility considerations in the perioperative setting.

Overall, although microbiota-focused interventions provide a useful conceptual framework for understanding gut-liver-brain interactions, their translation into perioperative strategies for POD prevention is limited by heterogeneous study designs, variability in interventions, and the absence of standardized delirium outcomes. Future research should prioritize well-powered, mechanism-informed randomized controlled trials integrating longitudinal microbiome profiling with validated delirium assessments in high-risk populations.

At present, no microbiota-targeted intervention can be recommended for routine POD prevention in patients with liver disease outside of clinical trials.
